# Kohorten in der psychiatrischen Forschung

**DOI:** 10.1007/s00115-020-01043-3

**Published:** 2021-03-09

**Authors:** Andreas Heinz, Kristina Adorjan, Tobias Banaschewski, Gunter Schumann, Michael Rapp

**Affiliations:** 1 Klinik für Psychiatrie und Psychotherapie, CCM, Charité – Universitätsmedizin Berlin, Charitéplatz 1, 10117 Berlin, Deutschland; 2 Klinik für Psychiatrie und Psychotherapie, LMU Klinikum, München, Deutschland; 3 Institut für Psychiatrische Phänomik und Genomik (IPPG), LMU Klinikum, München, Deutschland; 4 Center for International Health (CIH), LMU München, München, Deutschland; 5 Klinik für Psychiatrie und Psychotherapie des Kindes- und Jugendalters, Zentralinstitut für seelische Gesundheit, Medizinische Fakultät Mannheim, Universität Heidelberg, Mannheim, Deutschland; 6 PONS Zentrum, Charité Mental Health, Klinik für Psychiatrie und Psychotherapie, Campus Charité Mitte, Berlin, Deutschland; 7 Centre for Population Neuroscience and Stratified Medicine (PONS), ISTBI, Fudan University, Shanghai, China; 8 Sozial- und Präventivmedizin, Department Sport- und Gesundheitswissenschaften, Strukturbereich Kognitionswissenschaften, und Fakultät für Gesundheitswissenschaften Brandenburg, Profilbereich für Versorgungsforschung mit Schwerpunkt eHealth, Universität Potsdam, Potsdam, Deutschland

Psychische Erkrankungen entstehen in komplexer Interaktion zwischen genetischen Risikofaktoren, sozialen Kontextbedingungen und individuellen Erfahrungen [1]. Kohorten bieten eine einzigartige Möglichkeit, solche Interaktionen im Längsschnitt zu untersuchen, wenn sie hinreichend umfassend charakterisiert wurden. Zu den wichtigsten Parametern einer solchen Charakterisierung gehören neben genetischen Faktoren auch epigenetische Variabilität, Daten über individuelle Erfahrungen, wie z. B. Traumatisierungen, Lebensräume, Einkommen, Bildungsgrad sowie Auswirkungen sozialer Ausschließung oder Diskriminierung. Zusammen mit einer standardisierten Erhebung des Verhaltens unter verschiedenen experimentellen Bedingungen und seiner neurobiologischen Korrelate, z. B. durch Bildgebungsuntersuchungen oder psychophysiologische Messungen [2], kann ein umfassendes Bild der einzelnen Person in ihren für die Entwicklung psychiatrischer Erkrankungen relevanten Lebenszusammenhängen erstellt werden. Die Untersuchung solcher Kohorten ist jedoch aufwendig, da sie eine hinreichend repräsentative und ausreichend große Gruppe von Personen voraussetzt, die über einen möglichst langen Zeitraum erfasst wird. Frühe Traumatisierungen haben schwerwiegende Auswirkungen auf die psychische Gesundheit im Jugend- und Erwachsenenalter. Daher sollte zumindest für derartige Fragestellungen die Untersuchung bereits bei der Geburt beginnen oder sogar intrauterine Belastungsfaktoren wie Rauchen oder Alkoholkonsum miterfassen [3].

Neue Erkenntnisse liefern standardisierte Datenerhebungen in unterschiedlichen Kontinenten

Im vorliegenden Schwerpunktheft von *Der Nervenarzt* werden unterschiedliche Kohorten und die hier erhobenen Erfahrungen und Herausforderungen diskutiert. Dies ist von großer aktueller Relevanz, da in diesen Tagen die Auswahl der Standorte für ein Deutsches Zentrum für Psychische Gesundheit (DZPG) erfolgt. Die Gründung des DZPG bietet die außerordentliche Gelegenheit, bestehende prospektive Kohorten zu koordinieren und fortzuführen und gleichzeitig durch die Bildung neuer Kohorten wichtige Bereiche der Gesundheitsvorsorge, die bisher nicht hinreichend erforscht sind, zu stärken.

*Adorjan et al.* schildern in ihrem Beitrag „Neurogenetik der Schizophrenie: Erkenntnisse aus Studien basierend auf Datentausch und globalen Partnerschaften“ entsprechend Untersuchungen bezüglich des Auftretens und der Ausprägung schizophrener Psychosen. Von besonderer Bedeutung hier ist die Erhebung molekulargenetischer und umweltbezogener Daten in unterschiedlichen Kontinenten, die es auch erlaubt, Umweltfaktoren zu identifizieren, die außerhalb der relativ gut beforschten europäischen und amerikanischen Lebenswelten zur Entstehung psychotischer Erfahrungen beitragen [4].

In der Arbeit mit dem Titel „Kohortenstudien in der Kinder- und Jungendpsychiatrie“ werden von *Holz et al.* Entwicklungsaspekte von Kinder und Jugendlichen diskutiert. Die Autoren verweisen hier insbesondere auf internationale longitudinale Studien, die sie in einer exemplarischen Beschreibung mit der der Mannheimer Risikokinderstudie vergleichen. Letztere untersucht ihre Probanden seit über 30 Jahren vom Zeitpunkt der Geburt an und hat wichtige Erkenntnisse zur Bedeutung der sozioökonomischen Ressourcen, des Status und geschlechtsspezifischer Effekte für das Auftreten psychischer Probleme in bestimmten Altersperioden geliefert. Dazu passend beschreiben *Rapp et al.* Potenziale und Grenzen von Alterskohortenstudien für die Gerontopsychiatrie. Auch hier geht es um die Erfassung spezifischer Mechanismen der Entstehung psychischer Erkrankungen in einem bestimmten Altersabschnitt, aus denen sich dann Präventionsansätze für psychische Erkrankungen ableiten lassen. Hier besteht allerdings ein relativer Mangel an klinischen Kohortenstudien, sodass hier der künftige Forschungsbedarf besonders deutlich wird.

Über Satellitenortung lässt sich Verhalten im sozialen Kontext erfassen

Internationale Aspekte der Forschung, die bereits in der Arbeit von *Adorjan et al.* angeklungen sind, werden im Folgenden in den Arbeiten von *Heinz et al.* zur IMAGEN-Kohorte und von *Schumann et al.* zu populationsneurowissenschaftlichen Strategien zur Erhebung und Analyse klinischer und globaler Kohorten in den STRATIFY- und GIGA-Konsortien diskutiert. Die IMAGEN-Kohorte ist eine in Europa erhobene Gruppe von über 2000 jungen Menschen, die erstmals im Alter von 14 Jahren untersucht wurden und mittlerweile seit über 8 Jahren in weiteren Beobachtungszyklen nachuntersucht werden konnten. Am Beispiel der IMAGEN-Kohorte zeigen *Heinz et al.*, dass sich sowohl nosologieübergreifende wie krankheitsspezifische Störungen im Bereich der Belohnungserwartung und der damit verbundenen neurobiologischen Systeme nachweisen lassen, die für die Entstehung von Suchterkrankungen, aber auch für affektive Störungen und Psychosen relevant sind. *Schumann et al.* erläutern, wie derzeit durch internationale Standardisierung und Angleichung der Erhebungsinstrumente Kernbefunde aus der IMAGEN-Kohorte in unterschiedlichen Kontinenten überprüft, differenziert und erweitert werden können [5]. Von besonderer Relevanz sind hier neuartige Technologien, die wie beispielsweise die Satellitenortung das Verhalten der Probanden im jeweiligen sozialen Kontext direkt erfassen können. Hierbei ergeben sich allerdings komplexe datenschutzrechtliche Aspekte, die insbesondere dann von zusätzlicher Problematik sind, wenn die Untersuchungen in unterschiedlichen politischen Systemen stattfinden [6]. Auf diese ethisch wie datenschutzrechtlich relevanten Aspekte gehen die genannten Arbeiten vertieft ein.

Mit der internationalen Vernetzung von Kohortenstudien ergibt sich so erstmals die Möglichkeit, die Bedeutung sozialer Faktoren für die Entstehung und Aufrechterhaltung psychischer Erkrankungen in ihrem Wechselspiel mit genetischer Vulnerabilität und kulturellen Besonderheiten in verschiedenen Regionen der Welt zu untersuchen. Dadurch werden Erkenntnisse vertieft, wie auch auf Ebene sozialer Interventionen präventiv und therapieunterstützend gehandelt werden kann. Es bleibt zu hoffen, dass die genannten Themen eine zentrale Stellung im künftigen Deutschen Zentrum für Psychische Gesundheit einnehmen können.

Prof. Dr. Dr. Andreas Heinz

Dr. Kristina Adorjan

Prof. Dr. Dr. Tobias Banaschewski

Prof. Dr. Gunter Schumann

Prof. Dr. Dr. Michael Rapp
